# A Study on the Preparation of Regular Multiple Micro-Electrolysis Filler and the Application in Pretreatment of Oil Refinery Wastewater

**DOI:** 10.3390/ijerph13050457

**Published:** 2016-04-29

**Authors:** Ruihong Yang, Jianzhong ZHU, Yingliu Li, Hui Zhang

**Affiliations:** 1 Key Laboratory for Integrated Regulation and Resource Development on Shallow Lake of Ministry of Education, College of Environment, Hohai University, Nanjing 210098, China; rhyang123@126.com (R.Y.); liyingliu@em.jpu.edu.cn (Y.L.); zhhhhz1@gmail.com (H.Z.); 2 Department of Chemical Engineering, Yangzhou Polytechnic Institute, Yangzhou 225127, China

**Keywords:** micro-electrolysis, fillers, preparation, oil refinery wastewater

## Abstract

Through a variety of material screening experiments, Al was selected as the added metal and constituted a multiple micro-electrolysis system of Fe/C/Al. The metal proportion of alloy-structured filler was also analyzed with the best Fe/C/Al ratio of 3:1:1. The regular Fe/C/Al multiple micro-electrolysis fillers were prepared using a high-temperature anaerobic roasting method. The optimum conditions for oil refinery wastewater treated by Fe/C/Al multiple micro-electrolysis were determined to be an initial pH value of 3, reaction time of 80 min, and 0.05 mol/L Na_2_SO_4_ additive concentration. The reaction mechanism of the treatment of oil refinery wastewater by Fe/C/Al micro-electrolysis was investigated. The process of the treatment of oil refinery wastewater with multiple micro-electrolysis conforms to the third-order reaction kinetics. The gas chromatography–mass spectrometry (GC–MS) used to analyze the organic compounds of the oil refinery wastewater before and after treatment and the Ultraviolet–visible spectroscopy (UV–VIS) absorption spectrum analyzed the degradation process of organic compounds in oil refinery wastewater. The treatment effect of Fe/C/Al multiple micro-electrolysis was examined in the continuous experiment under the optimum conditions, which showed high organic compound removal and stable treatment efficiency.

## 1. Introduction

Oil refinery wastewater is one of the major forms of industrial wastewater, mainly coming from oil refining units, including dewatering and desalting in electric dewatering plants, direct distillation of crude oil, and cracking and distillation of heavy oil. Some fraction is a collection of slick oil, emulsified oil, dissolving organic matter, and salt in the integration of a multiphase system, with high oil content, high COD content, BOD_5_, sulfide, volatile phenol, suspended solids, and ammonia, nitrogen, *etc.* [1,2]. Oil refining wastewater treatment technology can be divided into physical, chemical, biological, and physicochemical treatment. Physical treatment includes gravity settling methods, flotation, filtration methods, hydro-cyclone separation, *etc.* Chemical treatment includes coagulation methods, advanced oxidation technologies, and coarse graining technology, *etc.* Biochemical methods mainly include the biochemical tower, biological filter, MBR, AO process, *etc.* [3,4,5]. Physical and chemical methods have adsorption, membrane separation, magnetic separation technologies, and electrochemistry methods, *etc.* [6,7,8,9]. Several innovative technologies for oil refining wastewater treatment emerged, such as ultrasound and dispersed nanoscale zero-valent iron particle coupling techniques [10]; Liu *et al.* used a gas-liquid-solid three-phase flow airlift loop bioreactor to treat oil refinery wastewater [11]; Shariati *et al.* have reported membrane sequencing batch reactors and the effect of hydraulic retention time on the performance and fouling characteristics of membrane sequencing batch reactors was researched [12]; Carlos *et al.* used coagulation-flocculation and flotation coupling techniques to treat petroleum refinery effluent, and the optimization of coagulation-flocculation and flotation parameters were obtained [13]; a three-dimensional electrode reactor was used to pretreat heavy oil refinery wastewater by Wei *et al.* [14]; Saien treated oil refinery wastewater with photo-catalytic degradation technology under mild conditions [15].

At present, the process of “oil separator-air flotation-biological” is the most common process applied in domestic refining wastewater treatment. Outside drainage in most refining enterprises could attain national standards. However, with the increasingly serious situation of environmental protection and strict pollution discharge standards, the common process has been difficult to meet the requirement of the drainage standard and the common process need to be reinforced. Nowadays the main research direction is to enhance pretreatment units before the biochemical system, such as removing part of the refractory organic matter, reducing the organic load of the biochemical system, improving the wastewater biochemical performance, and strengthening the system through advanced oxidation techniques [16,17].

Micro-electrolysis technology is a simple and effective electrochemical oxidation technolog, also called internal micro-electrolysis, the iron reduction method, zero-valent iron, *etc.* It was introduced to China in the 1980s, obtained the favor of experts and scholars, and it has been widely used in dyeing, chemical, pharmaceutical, coking, and other industrial wastewater treatment [18,19,20,21]. The principle is applied in the potential difference between iron and carbon particles, iron with low potential anodes, and carbon with the high potential cathodes. Iron and carbon in the electrolyte solution form countless macro-galvanic cells. Electrochemical reactions can cause flocculation, precipitation, adsorption, bridging, electroplating, and other synergies [16]. In recent years, the research and application of micro-electrolysis technology in refractory biodegradable organic wastewater treatment at home and abroad was popular. Fe/C micro-electrolysis was applied in denitrification for the coking wastewater [22,23]; Lai *et al.* applied micro-electrolysis to treat wastewater from acrylonitrile-butadiene-styrene (ABS) resin manufacturing [24]; Yang *et al.* have discussed the mechanism, kinetics and application of interior micro-electrolysis on enhanced activated sludge [25]; an internal electrolysis filter was also applied in mixed chemical wastewater treatment [26,27]. Nowadays, many scholars combine micro-electrolysis with other techniques to treat the refractory organic wastewater. Huang *et al.* applied anaerobic treatment coupled with micro-electrolysis (ATCM) in the treatment of anthraquinone dye wastewater [28]; Qin *et al.* applied micro-electrolysis coupled with the membrane bio-reactor (MBR) process in treatment of anthraquinone dye wastewater [29].

The traditional Fe/C micro-electrolysis uses an iron and activated carbon physical mixture as filler; the reaction efficiency is low and has engineering problems, such as easy blocking, hardening, and deactivation during application, which has severe limitations for large-scale applications [30]. Aimed to improve the reaction efficiency and solve the practical problems in the application of micro-electrolysis technology, we attempted to develop more efficient metal alloy-structured regular multiple micro-electrolysis fillers. Then, the regular multiple micro-electrolysis filler was applied in oil refinery wastewater treatment by batch experiments to explore the influence factors of multiple micro-electrolysis. The effect of micro-electrolysis in oil refinery wastewater treatment and the change of filler after a long continuously-running time were studied by continuous experiments.

## 2. Materials and Analytical Methods

### 2.1. Materials

Iron powders include iron over 98.0% (analytically pure); activated carbon powder (analytically pure); aluminum powders include aluminum over 99.0% (analytically pure); nickel powders include nickel over 99.5% (analytically pure); copper powders include copper over 99.5% (analytically pure); CH_2_Cl_2_ (analytically pure); and the correlative reagents for COD, BOD_5_, and pH determined. The wastewater was the effluent of a primary flotation tank obtained from a wastewater treatment plant of a refinery in China, the characteristics are shown in Table 1.

### 2.2. Analysis Methods

The COD and BOD_5_ were determined to use the COD analyzer (COD-571, Rex, Shanghai, China) and BOD_5_ analyzer (LB-50, LOOBO, Qingdao, China), respectively. The BOD_5_/COD index (B/C) was used to assess the wastewater biodegradability. The pH was measured by pHS-3C meter (Rex, Shanghai, China). NH_3_-N of the samples was determined according to standard methods [31]. The oil concentration was measured by ultraviolet spectrophotometer (UV-1801) (Benifen-Ruili, Beijing, China). The ultraviolet absorption spectrum scanned by ultraviolet spectrophotometer (UV-1801). The UV–VIS absorption spectrum of the oil refinery wastewater carried out in 10 mm quartz cuvettes and the UV–VIS spectra were recorded from 190 to 400 nm using deionized water as a blank. The morphology of fillers before and after use were characterized by a S-4800 II FE-SEM field emission scanning electron microscope (FE-SEM, 30 kV, Hitachi, Tokyo, Japan). GC–MS was used for organic compounds analysis. Prior to GC–MS determination, a 1000 mL sample was extracted using 20 mL CH_2_Cl_2_ three times under acidic conditions and three times under alkaline conditions, respectively. The six extracted layers were mixed, dehydrated, with anhydrous sodium sulfate and dried with the aid of a nitrogen flow. The residue was dissolved in 1.0 mL CH_2_Cl_2_ and 1 µL was injected into a Trace DSQ II GC–MS system (Thermo Electron, Massachusetts, USA) equipped with a DB-5 capillary column with an inner diameter of 0.25 mm and 30.0 m in length, vaporization temperature of 280 °C, and separator temperature of 280 °C. The GC column was operated in temperature-programmed mode at 80 °C for 10 min, raised at 5 °C·min^−1^ to 140 °C (held for 2 min), and then raised at 5 °C·min^−1^ to 280 °C (held for 10 min). Carrier gas was He, the pre-column pressure was 10 pa, split ratio 10:1, the sample quantity was 1 µL. EI mass spectrometry ionization mode, electron bombardment energy 70 eV, electron multiplier voltage 1145 V, the ion source temperature was 140 °C, the scanning time was 1 s, and the quality range of 50–500 amu. Analysis was undertaken with reference to the NIST 05 mass spectral library database. Quantitative analysis used the peak area normalization method [32].

The COD removal rate at any distillation time t, *R_COD_* was calculated through Equation (1):

(1) $$R_{COD}=\frac{\left(C_{0}-C_{t}\right)}{C_{0}}\times 100\%$$

where *C_0_* is the initial COD value of raw wastewater and *C_t_* is the concentration of COD of the effluents at any time t.

### 2.3. Experimental Setup

#### 2.3.1. Experimental Method

(1) Iron and activated carbon pretreatment. The iron powder was soaked for 10 min in the mass fraction of 5% dilute sulfuric acid to remove the surface oxide layer, washed with NaOH solution for 10 min, and rinsed clean with distilled water. The activated carbon was soaked for 24 h in the raw water to establish the pollutant adsorption saturation.

(2) Determination of the added metal type and proportion. 300 mL of oil refinery wastewater was mixed with 200 g filler (mass ratio of Fe/C was 2:1; mass ratio of Fe/C/added metal was 2:1:1, ingredients of filler were physically mixed). The reaction mixtures were incubated at pH 3 for 60 min. NaOH was added into saved supernatants to adjust pH to *ca.* 9–10. After sedimentation and filtration processes, COD values were measured.

(3) Preparation of regular multiple micro-electrolytic filler. The regular multiple micro-electrolysis filler with a metal alloy structure and includes iron powder, activated carbon, and other metal catalysts. The preparation process of regular multiple micro-electrolysis filler includes the following steps: first, iron powder, activated carbon, metal catalyst, and bentonite are mixed in proportion (Fe/Al/C is 3:1:1 and bentonite 15%, the proportion and ingredients are determined by the following experiment) and a certain amount of distilled water was added to the mixture. Second, the mixture was granulated and shaped. Third, the mixture was cured anaerobically under nitrogen at x °C for 4 h. Fourth, the sample was roasted under nitrogen for 4 h at 1000–1100 °C temperature, followed by subsequent cooling to room temperature and storing under nitrogen.

(4) Pretreatment of homemade regular multiple micro-electrolysis filler. The filler was soaked two hours in the mass fraction of 5% dilute sulfuric acid to remove the surface oxide layer, rinsed clean with distilled water; then, the filler was soaked two hours in the raw water to establish the pollutant adsorption saturation.

(5) Influence factors. Under the condition of the pH of acid, 300 mL oil refinery wastewater was reacted with 200 g homemade regular Fe/C/Al multiple micro-electrolysis filler for 100 min, supernatants were taken and their pH adjusted to *ca.* 9–10 with NaOH, then sedimentation and filtration was performed, and the COD values were measured.

(6) Continuous experiment. Adjust pH of raw water to the acid inlet of the reactor, which was filled with the pretreated homemade regular multiple micro-electrolysis filler, took a certain amount of water in the sampling mouth at a certain time, with NaOH adjusted the pH to *ca.* 9–10, then sedimentation and filtration was performed, and the COD, NH_3_-N, oil, and BOD_5_ values were determined.

#### 2.3.2. Experimental Apparatus

**Figure 1:**
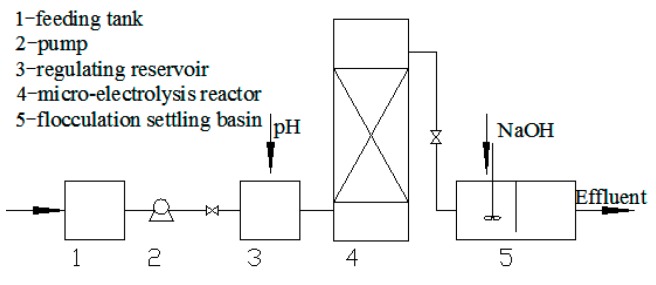
Continuous micro-electrolysis reactor.

The beaker batch experiments were used to study the influence factors of multiple micro-electrolysis. A continuous experiment was used to study the application of multiple micro-electrolysis. The device is shown in Figure 1. The reactor was made of a transparent synthetic glass column. The experimental apparatus is a cylindrical micro-electrolysis reactor (Ø 10 cm × 30 cm), the effective volume is about 2.0 L.

## 3. Results and Discussion

### 3.1. Materials Selection and Formula Test

The main ingredients of regular multiple micro-electrolysis fillers included iron, activated carbon, and additional metal. The key of the experiment was to select an additional metal type and determine the formula.

#### 3.1.1. The Types of Metal Catalysts Added

**Figure 2:**
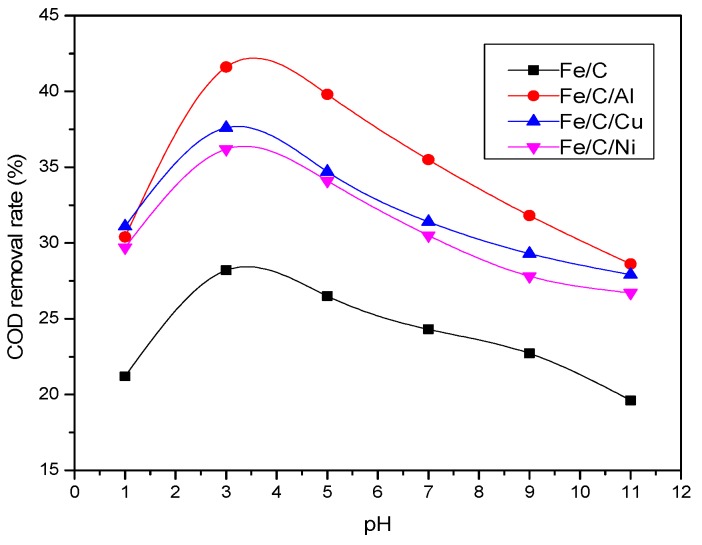
Influence of different metal catalysts on COD removal rate.

On the basis of traditional Fe/C micro-electrolysis, we added different kinds of metal catalysts such as Al, Cu, and Ni to constitute multiple micro-electrolysis systems to treat oil refinery wastewater. Through study of the treatment effect of multiple micro-electrolysis system, a suitable metal catalyst for oil refinery wastewater treatment was selected. The m(Fe/C) was 2:1; m(Fe/C/adding metal) was 2:1:1, pH were 1, 3, 5, 7, 9, and 11, and a reaction time of 60 min. The experimental results are shown in Figure 2.

Figure 2 showed, on the basis of the Fe/C system, added Cu, Ni, and Al to constitute the multiple micro-electrolysis system, respectively, which had an obvious difference in COD removal effects. The maximum COD removal rate of the Fe/C/Cu system was 37.6%, Fe/C/Ni system was 36.2%, and Fe/C/Al system was 41.6%. Visibly, the COD removal rates of all Fe/C/Cu, Fe/C/Ni, and Fe/C/Al systems were higher than the Fe/C system. Hence, adding Cu, Ni, and Al all has certain catalytic effects on micro-electrolysis. The highest COD removal rate was the Fe/C/Al system, so Al had the most obvious micro-electrolysis catalytic effect. Al was selected to be added to enhance the Fe/C micro-electrolysis system, constituting a Fe/C/Al multiple micro-electrolysis system. In addition, aimed at different kinds of wastewater, the choice of added metal catalyst types was different, and experiments were needed to determine the composition of effective components.

#### 3.1.2. Filler Formula Experiment

**Figure 3:**
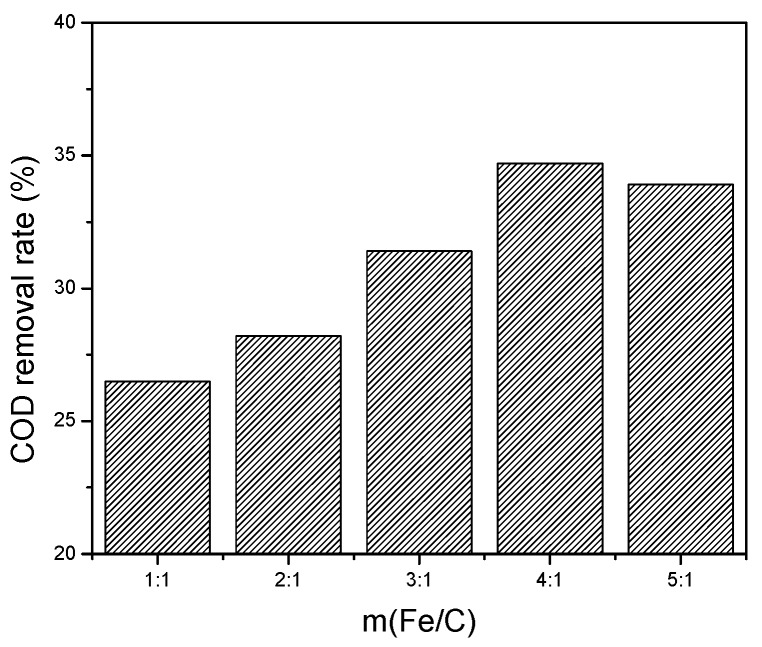
Influence of m(Fe/C) on the COD removal rate.

(1) m(Fe/C) proportioning experiment. Under the condition of pH 3, and 60 min reaction time, Fe/C micro-electrolysis on the COD removal rate is shown in Figure 3.

As shown in Figure 3, the COD removal rate reached a maximum of 34.7% when the m(Fe/C) was 4:1. When the m(Fe/C) was 4:1, the molar ratio was close to 1:1, it could constitute the highest number of micro-batteries in the micro-electrolysis system. When iron was deficient, it could not form enough micro-batteries, so the electrode reaction rate dropped, and COD removal rate would be low. When iron was in excess, the iron directly reacted with H^+^ to generate H_2_ and Fe^2+^, so it would generate less new ecological (H), the redox ability was weak, and the COD removal rate decreased. So, the reasonable m(Fe/C) was 4:1 when refining wastewater treatment by micro-electrolysis.

**Figure 4:**
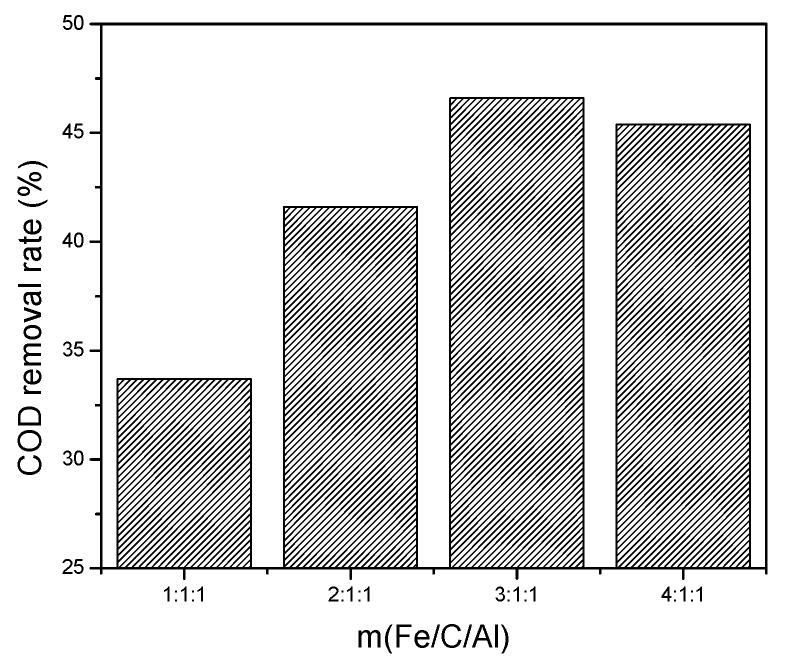
Influence of m(Fe/C/Al) on the COD removal rate.

(2) m(Fe/C/Al) proportioning experiment. Next, we discussed the influence of m(Fe/C/Al) on treatment efficiency in micro-electrolysis. The pH was 3–4, the m(Fe/C/Al) were 1:1:1, 2:1:1, 3:1:1, and 4:1:1, respectively, and the reaction time was 60 min. Influence of m(Fe/C/Al) on micro-electrolysis treatment effect is shown in Figure 4.

From Figure 4, the influence of different m(Fe/C/Al) on COD removal efficiency was obvious. When m(Fe/C/Al) was 3:1:1, COD removal rate increased up to 46.6%. The results indicated that the optimum m(Fe/C/Al) was 3:1:1 as the ratio of active ingredients of filler.

#### 3.1.3. The Physical and Chemical Characteristics of Multiple Micro-Electrolysis Filler

**Figure 5:**
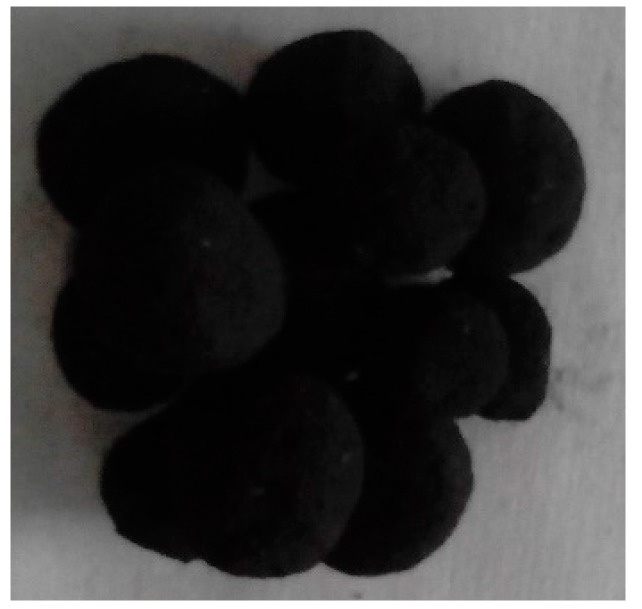
Shape of homemade regular Fe/C/Al multiple micro-electrolysis filler.

Regular filler was made by a high-temperature anaerobic roasting method as shown in Figure 5. It was sintered, forming a porous alloy spherical structure by reducing iron, aluminum, bentonite, and activated carbon. The bulk density of the filler was 1000–1100 kg/m^3^, porosity was 68%, specific surface area over 1.4 m^2^/g, and the size was 10 mm in diameter.

### 3.2. Influence Factors

#### 3.2.1. Influence of Initial pH value

**Figure 6:**
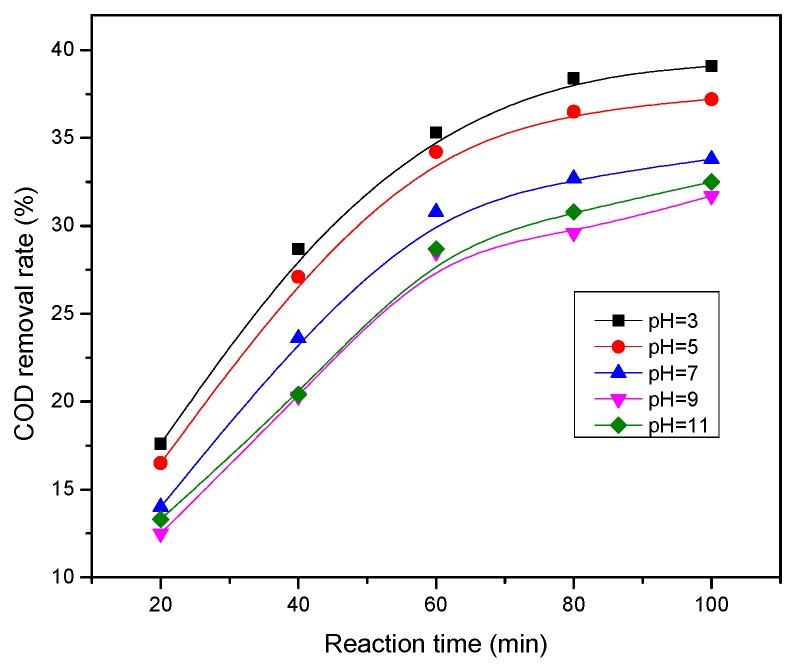
Influence of initial pH value on the COD removal rate.

These experiments were mainly to investigate micro-electrolysis treatment effects under different initial pH value conditions. The pH was 3, 5, 7, 9, and 11, respectively, and the reaction time was 100 min. The experimental results are shown in Figure 6.

Figure 6 showed the treatment effect was significantly different under the different initial pH values. When pH was 3, the COD removal rate was 39.1% after reacting for 100 min. The treatment effect significantly reduced with the increase of pH value when the pH exceeded 3. In solution with low pH, the galvanic cell reaction could be high, the anodic reaction produced new ecosystem bivalent iron, and the cathode produced new ecosystem (H), which could degrade many organic compounds in wastewater under acidic conditions. When pH was too low, the iron ion acid dissolution dominated, the electrochemical dissolution was less, and large amounts of hydrogen were produced quickly and reacted with the iron. The degradation of organic compounds generally occurred on the iron surface, thus hindering the organic pollutants’ contact with the solid surface. Under high pH conditions, iron and aluminum ions generated a complex which attached to the surface of the filler and hindered the efforts of the micro-electrolysis reaction [20]. Thus, the most proper pH value of multiple micro-electrolysis reaction was 3.

#### 3.2.2. Influence of Auxiliary Electrolyte Dosing

**Figure 7:**
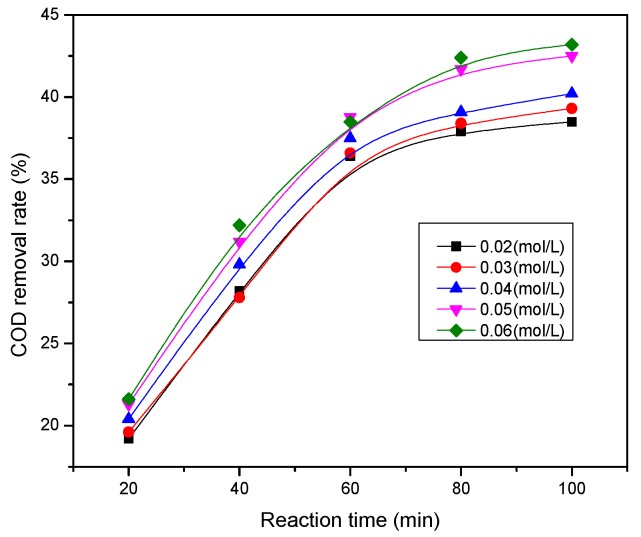
Influence of the concentration of auxiliary electrolyte on the COD removal rate.

This experiment was mainly to investigate the influence of auxiliary electrolyte dosing on the treatment effect. The pH was 3, auxiliary electrolyte Na_2_SO_4_ additive concentration was 0.02, 0.03, 0.04, 0.05, and 0.06 mol/L, respectively, and the reaction time was 100 min. The experiment results are shown in Figure 7.

As Figure 7 shows, the COD removal rate was improved when Na_2_SO_4_ was added as the auxiliary electrolyte to the reaction system with different concentrations. When the concentration of Na_2_SO_4_ was less than 0.05 mol/L, the COD removal rate of oil refinery wastewater increased with the increase of auxiliary electrolyte Na_2_SO_4_ concentration. When Na_2_SO_4_ concentration was 0.05 mol/L, the COD removal rate reached 42.5% after 100 min incubation, while continuously increasing the concentration of the auxiliary electrolytes would not significantly boost the processing efficiency. The main reason was the electrical conductivity of the reaction system which was improved with the increase of auxiliary electrolyte concentration. It enhanced the mass transfer rate and promoted the micro-electrolysis degradation of wastewater better [28].

### 3.3. The Comprehensive Treatment Effect under the Optimal Conditions

**Figure 8:**
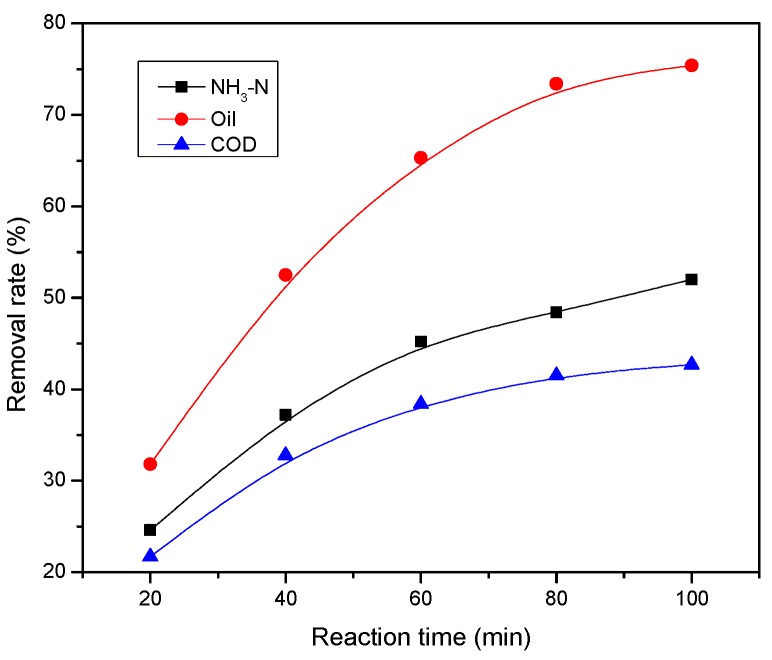
The comprehensive treatment effect under the optimum condition.

This experiment mainly studied the comprehensive treatment effect of refining wastewater treated by multiple micro-electrolysis technology under the optimum process conditions. The three major indicators, COD, NH_3_-N, and oil removal rate, were mainly investigated. The results are shown in Figure 8.

As Figure 8 shows, the treatment effect gradually improved with the extension of the reaction time. When the reaction time reached 80 min, the removal rate of COD was 41.5%, NH_3_-N 48.4%, and oil 73.4%. When the reaction time was continuously extended, the treatment effect had no significant improvement. The micro-electrolysis reaction would be fuller with a longer reaction time, but since the reaction progressed, H^+^ was consumed. If the H^+^ concentration reduced, the reaction rate significantly decreased and the reaction eventually stopped. Thus, there was no sense in prolonging the reaction time after 80 min.

### 3.4. Reaction Kinetics Study

The multiple micro-electrolysis reaction was carried out under the optimum reaction conditions. The COD values of refining wastewater were measured at the reaction time of 0, 20, 40, 60, 80, 100, and 120 min, respectively. The experiment results are shown in Table 2.

The treatment process of oil refinery wastewater was fitted by zero-order, first order, second-order, and third-order reaction kinetics based on the data of Table 2, respectively. The various levels reaction kinetics equations are obtained as indicated in Table 3.

From Table 3, when compared the correlation coefficient of zero-order, first order, second-order, and third-order reaction kinetics, we found the correlation coefficient of third-order reaction kinetics was the best with R^2^ = 0.9725. Thus, the process of multiple micro-electrolysis treated oil refinery wastewaters conformed to the third-order reaction kinetics, and the reaction rate constant was 1.4 × 10^−1^.

### 3.5. Continuous Running Experiment

#### 3.5.1. Continuous Running Effect

**Figure 9:**
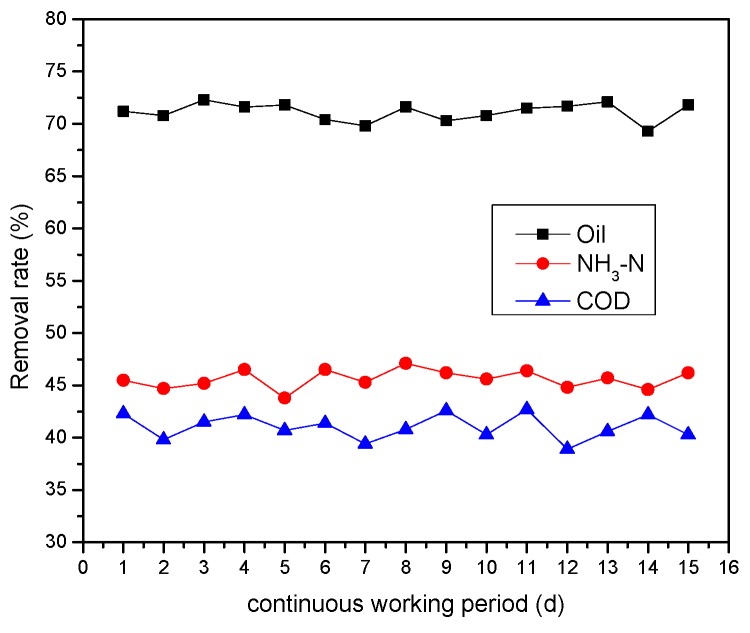
Removal efficiency of pollutants for continuous running.

(1) The removal efficiency of pollutants. These experiments mainly studied the treatment effect of oil refinery wastewater by multiple micro-electrolysis under the optimum condition (pH 3, supporting electrolyte concentration was 0.05 mol/L, hydraulic retention time of 80 min.) running continuously for 15 days. The running results are shown in Figure 9.

Figure 9 showed, with continuous running for 15 days, the COD removal rate of effluent steady at 39.4% to 42.7%, NH_3_-N removal rate steady at 43.8% to 46.5%, oil removal rate steady at 69.3% to 72.3%, and visible operation effect was stable. The regular filler was a metal alloy structured by high-temperature sintering, with a stable proportion of each component, and the contact between each composition was full. The regular alloy structure filler could ensure that the galvanic cell effect remained continuous and with high efficiency, and the separation of anode and cathode will not appear, like with physical mixing of the traditional filler [30]. The structure was formed with great specific surface area and uniform water circulation. The wastewater treatment provided a larger current density and better effect of catalytic reaction; the filler had a strong activity, and specific gravity was low, but lacked passivation and did not harden. The reaction rate was fast, and long-term operation was stable and effective.

**Figure 10:**
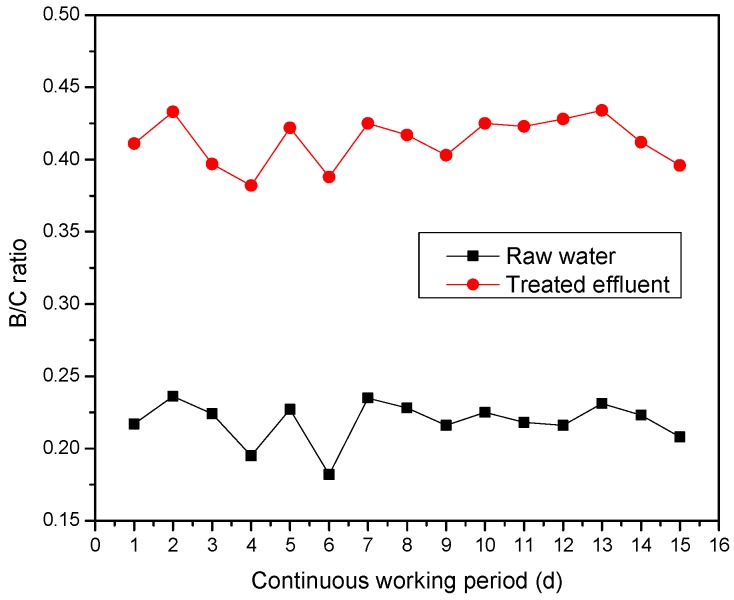
Comparison of B/C ratios before and after micro-electrolysis treatment.

(2) Biodegradability enhancement. Continuously working for 15 days, the water samples were taken at the inlet and outlet of the micro electrolysis reaction device and COD and BOD_5_ were determined; B/C ratio were computed, and the change of B/C ratio before and after micro-electrolysis reaction was studied. The results are shown in Figure 10.

Figure 10 showed that the B/C ratio of raw wastewater was 0.182 to 0.236, through multiple micro-electrolysis treatments the B/C ratio of effluent was steady at 0.382 to 0.434, the average B/C ratio of refining wastewater increased from 0.218 to 0.413, B/C ratio increased to 89.4%, benefit great to the subsequent biochemical treatment. There were two major reasons: on the one hand, a large number of ferrous ions were produced by anode and countless (H) and (OH) produced by cathode in the process of micro-electrolysis reaction, which could degrade the no biodegradable organic in oil refinery wastewater into small molecular and easily biodegradable organic, thus, it enhanced the biological availability; On the other hand, some refractory organics were adsorbed by the mix flocculation body of Fe(OH)_2_, Fe(OH)_3_ and Al(OH)_3_ and removed by co-precipitation in the flocculation sedimentation unit, thereby the hard biodegradable organic matter content in the wastewater was reduced. Visibly, multiple micro-electrolysis technology has obvious effect to improve the biological availability of oil refinery wastewater; pretreatment of the oil refinery wastewater with micro-electrolysis technology was advantageous to the enhancement of biochemical processing system [16].

#### 3.5.2. GC/MS Analysis of the Pollutions of Raw Wastewater and Effluent

**Figure 11:**
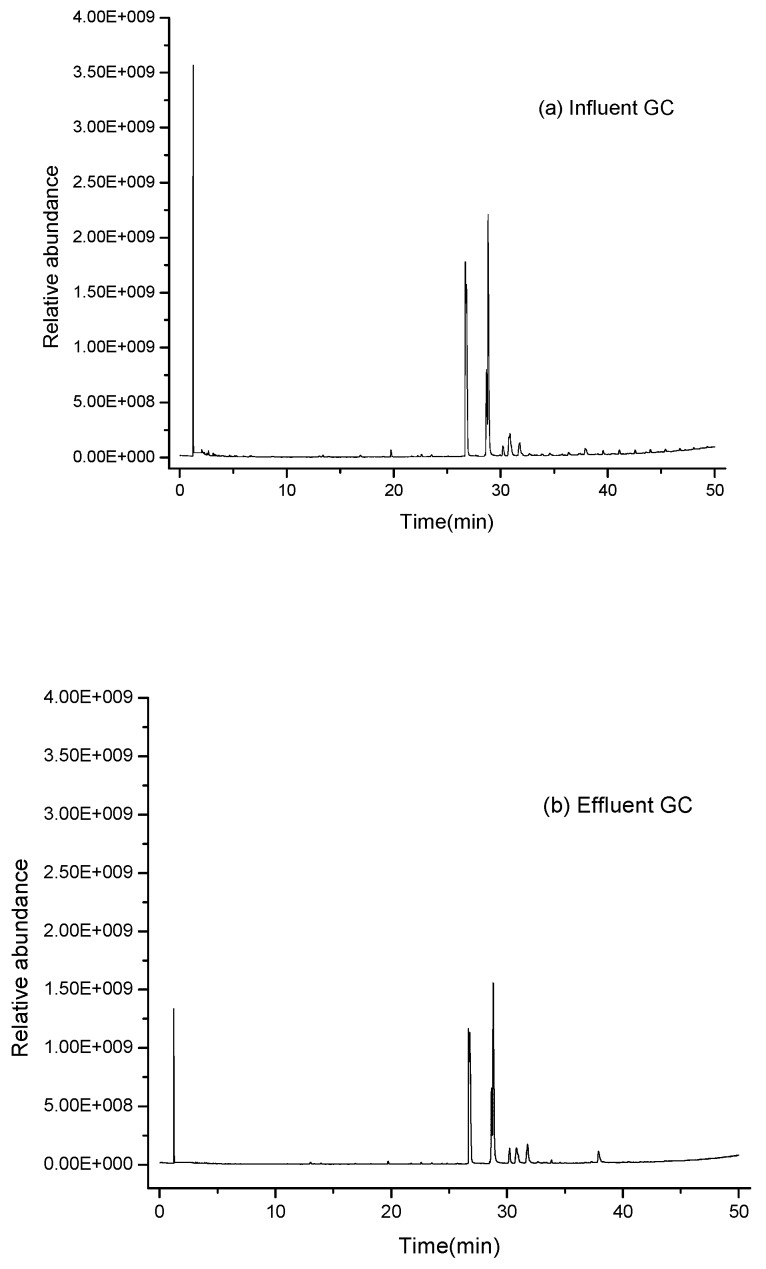
GC–MS chromatograms on dichloromethane extract from (**a**) influent and (**b**) effluent of the micro-electrolysis reactor.

GC–MS has been known for its superior separation of complex organic compounds, greater sensitivity, and shorter measuring time [8,24]. Thus, it was used to detect and identify the organic compounds in the oil refinery wastewater. Figure 11a and Table 4 show the GC and the organic compound distribution of the oil refinery’s wastewater. According to GC-MS analysis, the main organic compounds in the raw oil refinery’s wastewater are phenol, phenol,3-methyl, 1-hexene,3,4-dimethyl, phenol,3,4-dimethyl, and other complex organic matter, which contribute to the COD value and pollute the environment. From Figure 11a,b, it can be seen clearly that the organic compounds in the effluents were obviously reduced, which indicated that 1-hexene,3,4-dimethyl, phenol, phenol,3-methyl, and phenol,3,4-dimethyl could be removed from the water by micro-electrolysis in varying degrees.

#### 3.5.3. UV–VIS Spectral of the Degradation Process

**Figure 12:**
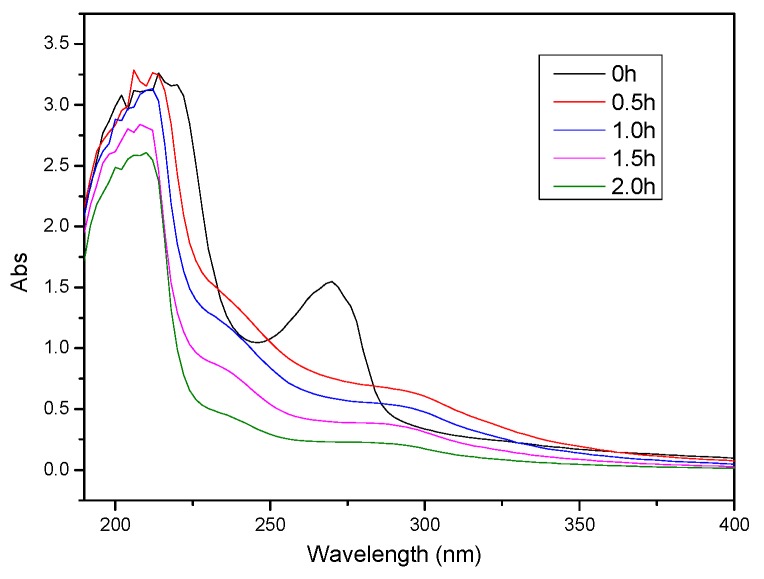
UV–VIS spectral change of oil refinery wastewater with different degradation times.

Petroleum and its products have characteristic absorption in the ultraviolet region. The main absorption wavelength of aromatic compounds with a benzene ring is 250–260 nm; the mainly-absorbed wavelength of compounds with conjugated double bonds is 215–235 nm. Figure 12 indicates that there are two obvious characteristic absorption peaks in the ultraviolet absorption spectrum of raw water in the wavelength of 215–235 nm and 250–260 nm. As the reaction proceeded, wastewater at each wavelength absorbance reduced which indicated that the concentration of organic matter in the wastewater decreased, but not obviously, and led to the COD removal rate of oil refinery wastewater treatment being only about 40%. In order to improve the efficiency of micro-electrolysis technology for refinery sewage treatment, on the one hand, more effective multiple micro-electrolysis fillers need to be developed while, on the other hand, micro-electrolysis technology or combination with other process also need to be strengthened.

#### 3.5.4. The Comparison of the Filler Change before and after Using

**Figure 13:**
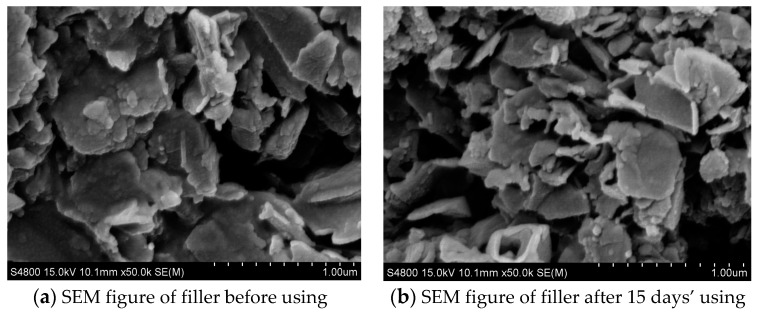
SEM contrast figure of filler before and after using. (**a**) SEM figure of filler before using and (**b**) SEM figure of filler after 15 days’ use.

The S-4800 II FE-SEM (FE-SEM, 30 kV, Hitachi, Tokyo, Japan) was used to observe the morphologies of fillers before and after using, the results are shown in Figure 13.

Figure 13 shows a SEM contrast figure of the filler before and after use. Comparing Figure 13b with Figure 13a, the morphology of the filler saw no significant change after 15 days’ use. The pore shape of the filler was intact, no jam phenomenon occurred, which showed that the alloy structure of the filler had high strength, good physical structure, and running wear resistance in the process of consumption, ensuring that the galvanic effect was continuously high. Thus, the quality of effluent was stable and the usable life was long.

### 3.6. Reaction Mechanism of Multiple Micro-Electrolysis

Fe and activated carbon can form galvanic cells in electrolyte solution [20,23]. The electrode reaction can be presented as follows:

(2) Anode: Fe−2e→Fe^2+^; E^ɵ^(Fe^2+^/Fe) = −0.44V

(3) Cathode: 2H^+^ + 2e→2[H]→H_2_↑; E^ɵ^(H^+^/H_2_) = 0.00V (Acidic)

When Al powder is added to the system, which also can form galvanic cells with activated carbon, the electrode reaction can be presented as follows [12]:

(4) Anode: Al−3e→A1^3+^; E^ɵ^(Al^3+^/Al) = −1.66V

(5) Cathode: 2H^+^ + 2e→2[H]→H_2_↑; E^ɵ^(H^+^/H_2_) = 0.00V (Acid)

**Figure 14:**
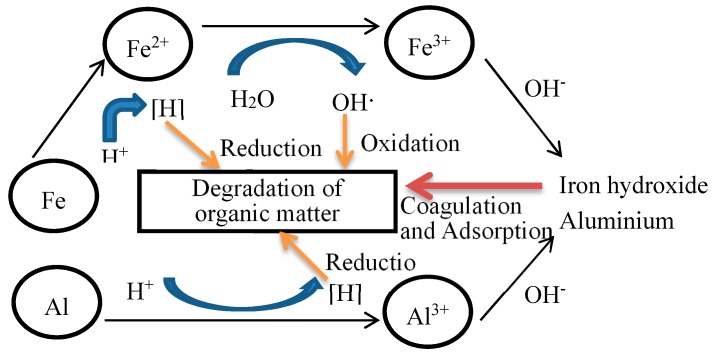
The reaction mechanism of Fe/Al/C multiple micro-electrolysis.

As can be seen from the above electrode reaction, in multiple micro-electrolysis systems Fe and C could form micro-batteries, Al and C could also form micro-batteries, and the conductivity of Al was superior to promote galvanic cell reaction. In multiple micro-electrolysis systems, Al and Fe constituted a bimetal catalytic system and improved the efficiency of the catalytic degradation reaction; the sediment of Fe and Al can form the synergy flocculation and settleability. Figure 14 shows the schematic diagram of the reaction mechanism of Fe/Al/C multiple micro-electrolysis.

As shown in Figure 14, in Fe/Al/C multiple micro-electrolysis system reactions, on the one hand, the anode reaction produced the new ecological bivalent iron ion which had strong reducing power, which could graduate part of the refractory ring and long chain organic into easily biodegradable small molecules of organic matter; the cathode reaction produced a large number of new ecological (H) and (OH), and could also react with many organic component of the wastewater. This resulted in organic macromolecular chain scission and decomposition to small-molecule organic matter. On the other hand, Fe^2+^, Fe^3+^, and A1^3+^ generated in the reaction, under alkaline pH of 9–10, could form Fe(OH)_2_, Fe(OH)_3_, and Al(OH)_3_. The synergy of Fe(OH)_2_, Fe(OH)_3_, and Al(OH)_3_ made the flocculation and settleability, which was superior to pure Fe(OH)_2_ and Fe(OH)_3_. Therefore, added Al could improve the removal efficiency of COD, but the Al additive quantity should not excessive, because the corrosion of aluminum in Fe/Al battery reactions can inhibit the corrosion of iron, thereby reducing the generation of ferrous ion.

## 4. Conclusions

(1) Al was determined as the adding metal composition of the micro-electrolysis filler, to constitute Fe/C/Al multiple micro-electrolytic systems. The new regular Fe/C/Al multiple micro-electrolysis filler was prepared, which was a granular structure of the metal alloy, and the optimum m(Fe/C/Al) was 3:1:1.

(2) The application effect of oil refinery wastewater pretreatment by Fe/C/Al multiple micro-electrolysis was remarkable. The optimum process parameters of pH 3, reaction time 80 min, Na_2_SO_4_ additive concentration 0.05 mol/L.

(3) When continuously running for 15 days, the COD, NH_3_-N, and oil removal rate were stable, the average B/C ratio of wastewater increased from 0.218 to 0.413, and the biodegradability of the wastewater had remarkably improved.

This study shows that the Fe/C/Al multiple micro-electrolysis can be considered as an effective and robust method for oil refinery wastewater pretreatment.

## Figures and Tables

**Table 1:** Characteristics of oil refinery wastewater.

Index	COD (mg/L)	BOD_5_ (mg/L)	NH_3_-H (mg/L)	Oil (mg/L)	pH
Value	340–430	76–95	7–15	32–48	6–8

**Table 2:** The concentration of COD of oil refinery wastewater at different times.

t/min	0	20	40	60	80	100	120
C (g/L)	0.365	0.287	0.253	0.235	0.216	0.208	0.201
lnC	−1.008	−1.248	−1.374	−1.448	−1.532	−1.570	−1.604
1/C	2.739	3.484	3.953	4.255	4.629	4.808	4.975
1/C^2^	7.506	12.140	15.623	18.108	21.433	23.114	24.752

**Table 3:** Equation of reaction kinetics for multiple micro-electrolysis degrading COD of oil refinery wastewater.

Level of Reaction Equation	Equation	Correlation Coefficient	Reaction Rate Constant
Zero-order	C = −0.0012t + 0.3258	R^2^ = 0.8379	1.2 × 10^−3^
First-order	−lnC = 0.0046t + 1.1203	R^2^ = 0.8949	4.6 × 10^−3^
Second-order	1/C = 0.0179t + 3.046	R^2^ = 0.9407	1.8 × 10^−2^
Third-order	1/C ^2^ = 0.142t + 9.0079	R^2^ = 0.9725	1.4 × 10^−1^

In the equations: C represents the COD concentration of refining wastewater after reaction time t, g/L; t represents the reaction time, min.

**Table 4:** Main component distribution of the raw oil refinery wastewater.

No.	Retention Time (min)	Compounds	Molecular Formula	Proposed Structures	Possible (%)	Area Percentage (%)
1	1.26	1-Hexene,3,4-dimethyl	C_8_H_16_	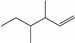	31.49	13.73
2	19.75	Naphthalene	C_10_H_8_	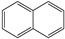	24.4	0.64
3	26.70	Phenol	C_6_H_6_O	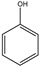	42.64	34.19
4	28.83	Phenol,3-methyl	C_7_H_8_O	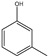	62.43	26.17
5	30.87	Phenol,3,4-dimethyl	C_8_H_10_O	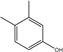	34.67	8.01
6	Other organic compounds					17.26
